# Increased investment in Universal Health Coverage in Sub–Saharan Africa is crucial to attain the Sustainable Development Goal 3 targets on maternal and child health

**DOI:** 10.1186/s13690-023-01052-z

**Published:** 2023-03-04

**Authors:** Robert Kokou Dowou, Hubert Amu, Farrukh Ishaque Saah, Oluwafemi Adeagbo, Luchuo Engelbert Bain

**Affiliations:** 1 Department of Epidemiology and Biostatistics, Fred N. Binka School of Public Health, University of Health, and Allied Science, Hohoe, Ghana; 2 Department of Population and Behavioural Sciences, Fred N. Binka School of Public Health, University of Health and Allied Science, Hohoe, Ghana; 3 Department of Community and Behavioral Health, College of Public Health, University of Iowa, Iowa City, USA; 4 Department of Sociology, Faculty of Humanities, University of Johannesburg, Johannesburg, South Africa; 5 Department of Psychology, Faculty of Humanities, University of Johannesburg, Johannesburg, Auckland Park, South Africa; 6 International Development Research Centre, IDRC, Ottawa, Canada

**Keywords:** Universal Health Coverage, Universal Health Monitoring Framework, Sub-Saharan Africa, Sustainable Development Goal, Maternal and child health

## Abstract

Universal Health Coverage (UHC) is considered a strategic component of the Sustainable Development Goals specifically for goal 3 which seeks to ensure healthy lives and promote well-being for all, where all individuals and communities have equal access to key promotive, preventive, curative, and rehabilitative health interventions without financial constraints. Despite Sub-Saharan Africa (SSA) accelerated gains on the UHC effective coverage of 2.6% between 2010 to 2019, many countries in the sub-region show lagging performance. The major challenges faced in attaining the UHC in many countries include inadequate capital investment for health and their equitable distribution, fiscal space to finance UHC policies and programs. This paper discusses how increased investment in Universal Health Coverage in SSA is crucial to attain the Sustainable Development Goal 3 targets on maternal and child health. The Universal Health Monitoring Framework (UHMF) is adopted in this paper as the underpinning framework. The delivery of essential maternal and child health services to achieve UHC in SSA requires strategic actions such as policies, plans and programs with focus on maternal and child health. We report findings from recently published papers that clearly highlighted the strong connection between health insurance coverage and maternal health care utilization. Strategic actions such as implementing national health insurance scheme (NHIS) that directly incorporates free maternal and child health care could strengthen maternal health services and transform health systems in order to achieve UHC in SSA. We argue that achieving the SDG 3 on maternal and child health will only be possible if significant progress in made in increasing UHC. This is key to ensure optimal maternal health care utilization, and consequently reducing maternal and child deaths.

## Systematic search method

We searched mainly google scholar, WHO Afro Library, WHO Global Index Medicus. MEDLINE, EMBASE, all literature published from 2015 to 2022. A combination of key text words headings such as “Universal Health Coverage, Universal Health Monitoring Framework, Sub-Saharan Africa, Sustainable Development Goal, maternal and child health” were used to identify literature on universal health coverage in SSA.

## Background

Achieving Universal Health Coverage (UHC) is one of the objectives in the Sustainable Development Goals (SDGs) agenda target three of improving maternal and child health by reducing the maternal mortality ratio to less than 70 per 100,000 live births and ending preventable to reduce neonatal mortality to at least as low as 12 per 1,000 live births and under-5 mortality to at least as low as 25 per 1,000 live births by 2030 [1]. UHC is considered a vital component of the SDGs specifically for goal 3 which seeks to ensure healthy lives and promote well-being for all [1, 2]. UHC is when all individuals and communities have equal access to key promotive, preventive, curative, and rehabilitative health interventions without financial constraints [3]. UHC is targeted at protecting people from the financial burden of paying for health services out of pocket and thus, reducing the risk of people being pushed into poverty due to unexpected illness that may require them to spend their life savings, and/or sell their assets to cover medical bills [4, 5].

Most of 48 of Sub-Saharan Africa (SSA) countries showed progressive gains on the UHC effective coverage since its initiation as a way to improve the health people especially maternal and child health [6]. Nevertheless, there are still many countries in the sub-region that show lagging performance on effective coverage of maternal and child health [7]. Nations in SSA have also committed and put measures in place to attaining the SDG declaration and their policies are directed towards principles of the UHC 2030 Compact, initiatives of UHC 2030 and the Political Declaration of UHC adopted at the UN High Level Meeting in September 2019 together with other principles including African Union Agenda 2063 [8, 9]. The common challenges faced in attaining the UHC in many countries include inadequate capital investment for health services and their equitable distribution, fiscal space to finance UHC policies and programs [10, 11].

While maternal mortality ratio declined globally by 44% from 1990 to 2015, the 75% reduction target was not met, and many countries especially in SSA fell short of achieving the Millenium Development Goal (MDG) 5 targets of reducing maternal mortality [12]. As indicated by WHO [12], there were still high maternal mortality with an estimated 303,000 maternal death annually where majority was found in SSA. After the expiration of the MDG in 2015, it was replaced with the 17 SDGs [13]. The commitment of ending preventable maternal mortality (EPMM), released by WHO in 2015 [14] as the MDG agenda was integrated into the maternal and child health target which was listed at the top of SDG target 3 [1]. Emphasis has therefore been placed on the importance of antenatal care to maternal and child survival [15]. However, in SSA, accessibility and coverage of essential maternal and child health services are low [16] especially with lowest antenatal care utilization among women [15].

Recent study by Bain, Aboagye, Dowou, Kongnyuy, Memiah and Amu [17] revealed that the prevalence of maternal healthcare utilisation among young women in SSA was 55.2%, 78.8%, and 40% for antenatal care (ANC), skilled birth attendance (SBA), and postnatal care (PNC) respectively. Similarly, Amu, Aboagye, Dowou, Kongnyuy, Adoma, Memiah, Tarkang and Bain [18] found that the prevalence of maternal healthcare utilisation in SSA was 58%, 70.6% and 40.7% for ANC, SBA and PNC, respectively. Poverty, poor health insurance coverage and remote place of residence from health facilities were shown by studies as the major determinant of maternal and childcare utilization by women in SSA. For instance, Amu et al. [18] noted that women covered by health insurance were 1.48, 1.37 and 1.42 more likely to utilise ANC, SBA and PNC and similarly indicated that women from rural settings were less likely to utilise maternal healthcare.

**Fig. 1:**
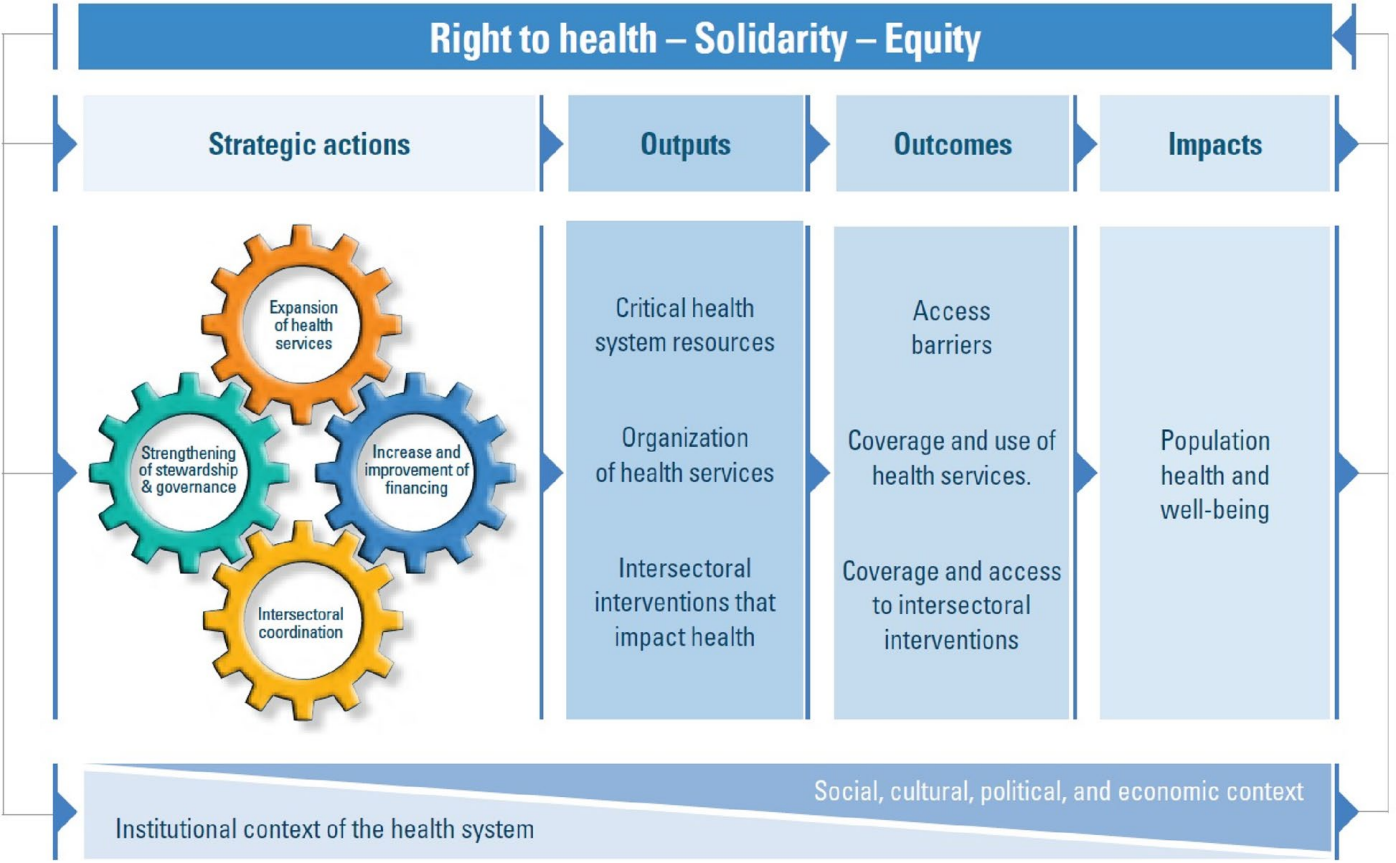
Universal Health Monitoring Framework. Source: PAHO (2021)

The Universal Health Monitoring Framework (UHMF) is adopted in this paper as the underpinning framework. The framework was adopted to underpinned this study because it to facilitates the analysis of progress and performance about public policies instituted to improve maternal and child health as way to achieve UHC by 2030 in SSA, the generation of evidence that could help in decision-making for the renovation or consolidation of health systems [19]. Also, UHMF forms an integral part of national processes of planning, monitoring, evaluation, and reporting of health system performance as a way in stimulation changes that contribute to improvement health outcome of people [19]. The framework has four dimensions including impacts, outcomes, outputs, and strategic actions. The dimension of UHMF help to track health status and well-being including maternal and child health. And also, it helps to measure progresses in universal access to health through a set of tracer indicators of magnitude and barriers to access to health services including maternal and child health services, use of health services, and access to intersectoral interventions that impact maternal and child health (Fig. 1).

The monitoring framework as adopted in this study describes the extent to which the health systems influence progress toward universal health. Strategic actions and outcomes are the areas in which the health sector has a greater degree of influence. In the context of universal health, strategic actions and their effect on outputs are the elements for which the health sector is largely responsible, although even these results are influenced by external factors and the health system context [19].

### Strategic actions

Strategic actions refer to health policies, interventions and programmes implemented by the government, health institutions and other stakeholders in an effort to strengthen or transform health systems with the goal of achieving universal health [20]. The strategic actions focus on improving comprehensive, quality, and community-centered health service. This tenet pinpoints the need to increase the stewardship and governance that could lead to political commitment and technical capacity building of healthcare providers geared towards promoting health system transformation aligned with the values of universal health [19]. Furthermore, the strategic actions involve increasing and improving financing with equity and efficiency and advancing toward the elimination of direct out-of-pocket payments by patients that mostly constitute a barrier to utilization of health service [20].

Universal access to maternal and child health and universal maternal and child health coverage is the two distinct however complementary bases of an equitable health system. Universal access to health services reduces all geographical, economic barriers [20]. Reiterated, the delivery of essential maternal and child health services to achieve UHC in SSA requires strategic actions such as policies, plans and programs with focus on maternal and child health. These actions are interdependent, hence the achievement of UHC results requires an integrated and comprehensive approach. It is essential for these processes to be integrated by governments in SSA to expand the coverage of critical system resources, ensure access to health services, and have an impact on health outcomes [19].

These strategic actions could prevent about two-thirds of child deaths, half to two-thirds of newborn deaths and many maternal deaths [21]. Investment of SSA countries in the expansion of equitable access to comprehensive and community centered health services could contribute to the improvement of maternal and child health leading to achieving SDG3. For example, Ghana implemented a Community-based Health Planning and Services (CHPS) that was based on task-shifting from institutional to outreach delivery of health services by training community health officers and volunteers to render home-based essential health services including life-saving maternal and child health services especially in rural communities [22–24]. This strategy has been reported to have contributed to an increased access and utilization of maternal and child health services in Ghana [25–27]. Similarly, implementation of the task shifting and sharing policy by the Nigerian Government through the training and capacity building and deployment community health workers, community health extension workers (CHEWs) and junior community health workers (JCHEWs) coupled with introduction of community midwifery at primary healthcare level have been crucial for promoting maternal, newborn and child health in Nigeria with consequence of reduction in the country’s maternal and neonatal mortality [28, 29].

Therefore, increasing investment in maternal and child health services, with equity and efficiency, and advancing toward the elimination of direct out-of-pocket payment and strengthening intersectoral coordination to address the social determinants of health is essential in achieving UHC. Strategic actions such as national health insurance scheme (NHIS) that directly incorporates free maternal and child health care could strengthen maternal health services or transform health systems in order to achieve UHC in SSA.

### Universal access and health coverage: outcomes and outputs

These constructs involve critical health system resources such as human resources for health, financing, and health technologies, organization of health services and intersectoral interventions that impact health [19, 20]. Despite of myriad of governmental and organizational efforts by various governments and international organization to improve the health outcome of women and their children over the last three decades, SSA continue to be one of the regions in the world where relatively maternal mortality ratios and under-5 mortality rates are still high [30]. Universal health coverage of maternal and child health through universal access to maternal and child health are prerequisite conditions for achieving health and well-being for all mother and their children require determining and implementing policies and actions with a multisectoral approach to address the issues in SSA [31].

To address the gap in universal access to maternal and child health care delivery, UHC was entrenched in SDGs with the target of providing financial risk protection by improving access and quality of healthcare delivery including improved access to safe, effective, quality, and affordable essential medicines and vaccines for all individuals [1]. Expanding coverage through access is necessary to improve people’s health status and overall well-being [32, 33].

### Impacts

The impact of health system is to promote health status and well-being of the population. Achieving this impact of universal health involves the formulation of innovative policies with action of political commitment that accelerate health systems transformation [20]. Maternal and child health issues have been at the center of international discourse over the past five decades. Maternal and child health status and wellbeing of all is central to achieving the 2030 Agenda for Sustainable Development Goals to which some SSA countries are signatories [1]. Major objectives of policies and interventions are to improve the health and wellbeing of mothers and their children focus on the determinants and systems that operate and maintain the health, safety, well-being, and appropriate development of children and their families in countries and societies in order to enhance the future health and welfare of societies and subsequent generations [34, 35]. Hence, UHC and maternal and child health have been linked together and remain a key area of focus in the third of the SDGs that relate to health [1, 36].

The SDGs recognize the significance of women's and children's health by adopting indicators for Goal 3 achievement which focused specifically on women's and children's health [36]. The first three indicators of the SDG three relate to maternal mortality, skilled delivery care, and the preventable deaths of newborns and children under five [37]. In other words, this means coverage of essential services based on interventions that include reproductive, maternal, newborn and child health, infectious diseases, non-communicable diseases and service capacity and access, at population level especially the underserved population [38].

The delivery of maternal and child health services in all settings requires adequate, competent, and motivated health workers with optimal skills mix at facility, outreach, and community level, and who are equitably distributed, adequately supported and enjoy decent work conditions [4]. However, there is a relative neglect for prioritizing investment in women’s and children’s health in the vision for primary health care thus the efforts to achieve UHC becomes questionable in achieving the health for all agenda [35]. This situation calls for immediate increase investment in training and deployment of qualified health professionals to all areas to render essential services to mothers and children [39].

## Conclusion

Maternal and child health are central in the achievement of the global SDG agenda which also prioritises the achievement of universal health coverage by the year 2030. Due to some challenges that inhibit access to maternal and child health services in SSA, accelerating progress towards the achievement of UHC and SDG target 3 ensures that preventable maternal and child mortalities are curtailed. National governments and international organizations must prioritise and ensure adequate access to health services especially in rural areas through increase investment in health infrastructure, logistics, training and deployment qualified health professionals to the last miles which are closest to the rural women. Resources needed for the efficient delivery of maternal and child health services such as essential medicines (e.g., ergometrine and Sulphadoxine Paramitamine) maternity beds, and vaccines for timely child immunization should be of priority in the health systems in SSA.

## Data Availability

The original contributions presented in the study are included in the article/supplementary material, further inquiries can be directed to the corresponding author.

## Abbreviations

UHMF: Universal health monitoring framework
UHC: Universal health coverage
SDG: Sustainable development goals
SBA: Skilled birth attendance
PNC: Postnatal care
ANC: Antenatal care
MDG: Millenium development goal
WHO: World health organization
NHIS: Health insurance scheme
SSA: Sub-Saharan Africa

## References

[1] Nations United (2015). Transforming our world: The 2030 agenda for sustainable development.

[2] Howden-Chapman P, Siri J, Chisholm E, Chapman R, Doll CN, Capon A. SDG 3: Ensure healthy lives and promote wellbeing for all at all ages. A guide to SDG interactions: from science to implementation. Paris: International Council for Science; 2017:81–126. https://council.science/wp-content/uploads/2017/03/SDGs-interactions-3-healthy-lives.pdf.

[3] Sanogo ND, Fantaye AW, Yaya S. Universal health coverage and facilitation of equitable access to care in Africa. Front Public Health. 2019;26(7):102. 10.3389/fpubh.2019.00102 PMC6497736 31080792

[4] Derkyi-Kwarteng AN, Agyepong IA, Enyimayew N, Gilson L (2021). A narrative synthesis review of out-of-pocket payments for health services under insurance regimes: a policy implementation gap hindering universal health coverage in sub-Saharan Africa. Int J Health Policy Manag.

[5] Hogan DR, Stevens GA, Hosseinpoor AR, Boerma T (2018). Monitoring universal health coverage within the Sustainable Development Goals: development and baseline data for an index of essential health services. Lancet Glob Health.

[6] Lozano R, Fullman N, Mumford JE, Knight M, Barthelemy CM, Abbafati C, Abbastabar H, Abd-Allah F, Abdollahi M, Abedi A, Abolhassani H (2020). Measuring universal health coverage based on an index of effective coverage of health services in 204 countries and territories, 1990–2019: a systematic analysis for the Global Burden of Disease Study 2019. The Lancet.

[7] Ministry of Health, Ghana. Ghana’s Roadmap for attaining Universal Health Coverage. 2019. Retrieved from: https://www.moh.gov.gh/wp-content/uploads/2021/08/UHC-Roadmap-2020-2030.pdf.

[8] Koomson I, Abdul-Mumuni A, Abbam A (2021). Effect of financial inclusion on out-of-pocket health expenditure: empirics from Ghana. Eur J Health Econ.

[9] Behera DK, Dash U (2019). Effects of economic growth towards government health financing of Indian states: an assessment from a fiscal space perspective. J Asian Public Policy.

[10] World Health Organization (2016). Fiscal space, public financial management, and health financing: sustaining progress towards UHC: implementation of the collaborative agenda, 26–28 April 2016, Montreux.

[11] World Health Organization (2015). Trends in maternal mortality: 1990–2015: estimates from WHO, UNICEF, UNFPA.

[12] Anand A, Roy N (2016). Transitioning toward sustainable development goals: The role of household environment in influencing child health in Sub-Saharan Africa and South Asia using recent demographic health surveys. Front Public Health.

[13] World Health Organization. Strategies towards ending preventable maternal mortality (EPMM); 2015b. Retrieved from https://apps.who.int/iris/bitstream/handle/10665/153544/9789241508483_eng.pdf.

[14] UNICEF. Unicef data: Antenatal care; 2021. Retrieved from: https://data.unicef.org/topic/maternal-health/antenatal-care/.

[15] Sanogo NA, Fantaye AW, Yaya S. Chapitre 5. Au-delà de la couverture: une étude qualitative explorant l’impact perçu du régime d’assurance maladie obligatoire du Gabon sur la qualité des soins prénatals Published in BMC Health services research. La couverture sanitaire universelle: Effet de l’assurance maladie obligatoire sur la qualité et l’accessibilité aux soins de santé au Gabon. BMC Health Serv Res. 2020;105(20):483. 10.1186/s12913-020-05310-6.

[16] Bain LE, Aboagye RG, Dowou RK, Kongnyuy EJ, Memiah P, Amu H (2022). Prevalence and determinants of maternal healthcare utilisation among young women in sub-Saharan Africa: cross-sectional analyses of demographic and health survey data. BMC Public Health.

[17] Amu H, Aboagye RG, Dowou RK, Kongnyuy EJ, Adoma PO, Memiah P, Tarkang EE, Bain LE. Towards achievement of Sustainable Development Goal 3: multilevel analyses of demographic and health survey data on health insurance coverage and maternal healthcare utilisation in sub-Saharan Africa. Health. 2022 Apr 19. International Health. 2022;0:1-16. 10.1093/inthealth/ihac017. 10.1093/inthealth/ihac017 PMC9977256 35439814

[18] Pan American Health Organization. Monitoring Framework for Universal Health in the Americas. Washington, D.C, Pan American Health Organization, 2021. Retrieved from http://www.paho.org/permissions on 14/03/2022.

[19] Bascolo E, Houghton N, del Riego A (2018). Construction of a monitoring framework for universal health. Rev Panam Salud Publica.

[20] Sheehan P, Sweeny K, Rasmussen B. Investing in Maternal, Newborn and Child Health: Analysis of the Costs, Benefits and Returns. 2012. Retrieved from: https://www.vu.edu.au/sites/default/files/cses/pdfs/2012-CSES-WHO-Investing-in-MNCH.pdf?_gl=1*cyymgv*_ga*MzI0OTM0NjkxLjE2Nzc4NDgzNjg.*_ga_Q1LS42WZC4*MTY3Nzg0ODM3Mi4xLjEuMTY3Nzg0ODM4My4wLjAuMA.

[21] Wright KJ, Biney A, Kushitor M, Awoonor-Williams JK, Bawah AA, Phillips JF (2020). Community perceptions of universal health coverage in eight districts of the Northern and Volta regions of Ghana. Glob Health Action.

[22] Awoonor-Williams JK, Bawah AA, Nyonator FK, Asuru R, Oduro A, Ofosu A, Phillips JF (2013). The Ghana essential health interventions program: a plausibility trial of the impact of health systems strengthening on maternal & child survival. BMC Health Serv Res.

[23] Strachan D, Cairncross S, Korkor AS, Hill Z (2012). Motivations and challenges of community-based surveillance volunteers in the northern region of Ghana. J Community Health.

[24] Haruna U, Dandeebo G, Galaa SZ (2019). Improving access and utilization of maternal healthcare services through focused antenatal care in rural Ghana: a qualitative study. Adv Public Health.

[25] Sakeah E, Aborigo R, Sakeah JK, Dalaba M, Kanyomse E, Azongo D, Anaseba D, Oladokun S, Oduro AR (2018). The role of community-based health services in influencing postnatal care visits in the Builsa and the West Mamprusi districts in rural Ghana. BMC Pregnancy Childbirth.

[26] Novignon J, Ofori B, Tabiri KG, Pulok MH (2019). Socioeconomic inequalities in maternal health care utilization in Ghana. Int J Equity Health.

[27] Doctor HV, Nkhana-Salimu S, Abdulsalam-Anibilowo M (2018). Health facility delivery in sub-Saharan Africa: successes, challenges, and implications for the 2030 development agenda. BMC Public Health.

[28] Adebajo S, Okereke E, Joseph F. Enhancing frontline health workers' abilities to improve MNCH services in Cross River State through task shifting/sharing. 2017. Retrieved from: https://knowledgecommons.popcouncil.org/departments_sbsr-rh/590/.

[29] Okereke E, Ishaku SM, Unumeri G, Mohammed B, Ahonsi B (2019). Reducing maternal and newborn mortality in Nigeria—a qualitative study of stakeholders’ perceptions about the performance of community health workers and the introduction of community midwifery at primary healthcare level. Hum Resour Health.

[30] Pan American Health Organization. Strategy for Universal Access to Health and Universal Health Coverage. 53rd Directing Council of PAHO, 66th Regional Committee of the World Health Organization for the Americas. Washington, D.C.: PAHO, 2014. Access on http://www.paho.org/uhexchange/index.php/en/uhexchange-documents/informacion-tecnica/27estrategia-para-el-acceso-universal-a-lasalud-y-la-cobertura-universal-de-salud/file on 18/03/2022.

[31] Boerma T, Requejo J, Victora CG, Amouzou A, George A, Agyepong I, Barroso C, Barros AJ, Bhutta ZA, Black RE, Borghi J (2018). Countdown to 2030: tracking progress towards universal coverage for reproductive, maternal, newborn, and child health. The Lancet.

[32] Khusakunrat P, Sriratanaban J (2017). Economic impact of investment in maternal and newborn health care under the National Health Security Scheme of Thailand. Asian Biomedicine.

[33] Ehiri J (2009). Maternal and Child Health: Global challenges, programs, and policies.

[34] Bustreo F, Doebbler C (2019). Universal Health Coverage: Are we losing our way on women’s and children’s health?. Health Hum Rights.

[35] Peters DH, Garg A, Bloom G, Walker DG, Brieger WR, Rahman MH (2008). Poverty and access to health care in developing countries. Ann N Y Acad Sci.

[36] UN General Assembly. Transforming our world: the 2030 Agenda for Sustainable Development; 2015. Retrieved from: https://www.un.org/en/development/desa/population/migration/generalassembly/docs/globalcompact/A_RES_70_1_E.pdf.

[37] UN General Assembly (2017). 71/313. Work of the Statistical Commission pertaining to the 2030 Agenda for Sustainable Development. Retrieved from: https://digitallibrary.un.org/record/1291226?ln=en#record-files-collapse-header.

[38] ten Hoope-Bender P, de Bernis L, Campbell J, Downe S, Fauveau V, Fogstad H, Homer CS, Kennedy HP, Matthews Z, McFadden A, Renfrew MJ (2014). Improvement of maternal and newborn health through midwifery. The Lancet.

[39] Dadjo J, Ahinkorah BO, Yaya S (2022). Health insurance coverage and antenatal care services utilization in West Africa. BMC Health Serv Res.

